# Imaging Multimodalities for Dissecting Alzheimer's Disease: Advanced Technologies of Positron Emission Tomography and Fluorescence Imaging

**DOI:** 10.3389/fnins.2015.00482

**Published:** 2015-12-22

**Authors:** Masafumi Shimojo, Makoto Higuchi, Tetsuya Suhara, Naruhiko Sahara

**Affiliations:** Molecular Neuroimaging Program, Molecular Imaging Center, National Institute of Radiological SciencesChiba, Japan

**Keywords:** Alzheimer's disease, biomarkers, multimodality, PET, fluorescence

## Abstract

The rapid progress in advanced imaging technologies has expanded our toolbox for monitoring a variety of biological aspects in living subjects including human. *In vivo* radiological imaging using small chemical tracers, such as with positron emission tomography, represents an especially vital breakthrough in the efforts to improve our understanding of the complicated cascade of neurodegenerative disorders including Alzheimer's disease (AD), and it has provided the most reliable visible biomarkers for enabling clinical diagnosis. At the same time, in combination with genetically modified animal model systems, the most recent innovation of fluorescence imaging is helping establish diverse applications in basic neuroscience research, from single-molecule analysis to animal behavior manipulation, suggesting the potential utility of fluorescence technology for dissecting the detailed molecular-based consequence of AD pathophysiology. In this review, our primary focus is on a current update of PET radiotracers and fluorescence indicators beneficial for understanding the AD cascade, and discussion of the utility and pitfalls of those imaging modalities for future translational research applications. We will also highlight current cutting-edge genetic approaches and discuss how to integrate individual technologies for further potential innovations.

## Introduction

Progressive neuronal loss and dysfunction in specific brain circuits are common neurological features of neurodegenerative disorders. The fundamental mechanism of neurodegenerative onset is still largely unknown, but accumulating evidence has indicated that deposition of filamentous and/or soluble oligomeric aggregates of specific proteins with significant neurotoxicity generally serves as the initial trigger for the sequential cascade of disease-related pathophysiology. In brain of Alzheimer's disease (AD) patients, senile plaques (SPs), and neurofibrillary tangles (NFTs) have been defined as two major pathological hallmarks, suggesting that the formation process of the resulting abnormal lesions is tightly linked to the pathogenic mechanism of AD. SPs are extracellular deposits composed of hydrophobic 38–49 amino acid peptides termed amyloid β (Aβ) and, since the discovery of the *in vitro* neuronal toxicity of aggregated Aβ species, numerous studies have indicated that brain accumulation of Aβ aggregates is a major causative risk factor for AD pathogenesis (Selkoe, 1991, 2001; Hardy and Selkoe, 2002). This “amyloid cascade hypothesis” is also strongly supported by the fact that Aβ metabolism is genetically linked to the familial AD causative genes β-amyloid precursor protein (APP) and Presenilins (PSs), adding major impetus to diverse therapeutic attempts to attenuate brain Aβ abnormality over the last two decades. However, in spite of these various advances in knowledge, no therapeutic approaches targeting Aβ have as yet successfully survived a Phase III trial (Huang and Mucke, 2012). This situation has now brought us to a turning point, as we need to re-evaluate our understanding of the consequences of AD pathophysiology and to carefully consider the direction of future therapeutic approaches so as to finally gain advantage over these complicated neurological disorders.

NFTs are composed of intra-neuronal and glial inclusions of hyperphosphorylated microtubule-associated protein Tau (MAPT), and they are widely observed in diverse tauopathies including progressive supranuclear palsy (PSP) and corticobasal degeneration (CBD), suggesting their importance for general neurodegenerative abnormalities (reviewed in Lee et al., 2001). In the pathological course of AD, NFTs begin to appear in the entorhinal cortex and then spatially spread through a wide range of brain areas including the hippocampus, limbic system, and neocortex (Braak and Braak, 1991; Braak et al., 2011). The severity of NFT lesions is well-correlated with synaptic dystrophy and neuronal loss, followed by brain atrophy, and test results of cognitive and memory impairment also revealed correlation with brain accumulation of tau (Arriagada et al., 1992). Importantly, pathological mutations in the genetic locus of *Tau* gene have been identified in the family of frontotemporal dementia with Parkinsonism linked to *MAPT* on chromosome-17 (FTDP-17-*MAPT*) (Hutton et al., 1998; Poorkaj et al., 1998; Spillantini et al., 1998). Furthermore, recent studies have demonstrated that transgenic mouse models overexpressing human Tau transgene harboring pathogenic FTDP-17-*MAPT* mutations exhibit robust NFTs formation followed by neuronal loss and memory impairment (reviewed in Sahara et al., 2011). These findings suggest that abnormal aggregation and/or physiological malfunction of Tau protein has more potential significance for neurodegeneration, and indicate that the severity of tau pathology is also a potent biomarker for the early clinical diagnosis of AD.

As proposed in Braak's model, the pathological severities of SPs and NFTs in post-mortem AD brains can be categorized into progressive stages (stages A-C for SPs and I-VI for NFTs), providing fundamental criteria for a definitive diagnosis of AD (Braak and Braak, 1991, 1997). Hopeful attempts by AD researchers to establish visible biomarkers for tracing the real time course of Aβ and tau lesions in brain of living patients have followed a long, sometimes frustrating journey, as so far, histological findings at autopsy have usually suffered from technical limitations during the post-mortem procedure. In this regard, an *in vivo* imaging approach is particularly important for assessing the consequence of clinical symptoms and spatial dynamics of pathological lesions simultaneously. For this purpose, non-invasive neuroimaging techniques represented by positron emission tomography (PET) and magnetic resonance imaging (MRI) have a major significance, as they allow us to monitor both functional and structural circumstances in brain of AD patients (Mori et al., 2012; Jack and Holtzman, 2013). Aβ PET radiotracers have been developed and approved for clinical use, and they have added in shedding more light on the process of the early detection of AD (Yang et al., 2012). More recently, several laboratories including our group demonstrated the potential utility of tau-specific PET radiotracers to validate the spatiotemporal kinetics of the pathological stage of NFTs (reviewed in Ariza et al., 2015; Villemagne et al., 2015). These consistent advances of radiological imaging technologies are really just a beginning stage, and further accumulations of new insights and important knowledge for constructing biomedical databases will be required for future translational research. It is important to realize that there is still a lot of room to manipulate and improve the capability of PET radiotracers in terms of binding specificity and chemical properties that can critically improve the diagnostic interpretation of PET neuroimaging. To visualize the brain status of AD more precisely, complimentary imaging approaches will be essential for assessing the detailed kinetics and fast dynamics of target molecules. Fluorescence-based imaging has great potential as a key technology to satisfy this aim. Although the utility of fluorescence is still restricted to animal models at this time, the major advantages of the fluorescence system are a variety of applications based on a wide range of probe design strategies including molecular genetics for dissecting the detailed molecular pathophysiology of neurodegenerative diseases. In this review, we will specifically introduce current imaging multimodalities beneficial for understanding the AD cascade and discuss how to integrate the respective modalities to the view of providing significant advances toward future translational research.

## PET imaging for diagnosis of alzheimer's disease

### General usage for brain PET imaging

Radiological techniques supply a variety of applications for *in vivo* brain imaging, becoming a most effective approach in terms of the clinical diagnosis of neurological disorders. PET technology is especially adept at monitoring the behavior of neuronal molecules in living subjects including rodent, monkey, and human. Although the spatial resolution obtained by MRI, which is superior for morphometric analysis, is much higher than that of PET imaging, the performance of PET is greatest in the quantification of the kinetics of neuronal receptor molecules and chemical probe interaction in intact brain. In principle, chemical probes are labeled by positron emitting radionuclides such as ^11^C and ^18^F, and PET scan identifies the spatial location of these probes as radiotracers with high detection sensitivity and superior quantitative dynamic range (Piel et al., 2014). Therefore, we can directly obtain a densitometric 3D map of the accumulation level of radiotracers binding to targets. A number of small chemical compounds with high affinity to target molecules can be screened as potential candidates of PET radiotracers without disrupting their parental structure and physicochemical properties. Only a small amount of radiation emitted from the tracer can be enough to allow detection, without any diffusion, and relatively fast decay of the radioisotope (*t*_1∕2_ = ~20 min for ^11^C, ~110 min for ^18^F) minimizes the radiation risk for patients (Piel et al., 2014). Furthermore, PET scan also provides important information about the detailed disposition and tissue circulation of the radiotracers themselves in brain, facilitating the design and synthesis of effective chemical derivatives that can successfully pass through the blood brain barrier (BBB) and penetrate brain tissue without obvious neurotoxicity (Piel et al., 2014). This basic feature of PET imaging makes this technology ideal for clinical use, and it also provides various potential utilities for scientific research.

### PET tracers for detecting Aβ and tau pathologies

Since the initial discovery of the β-sheet enriched common structure in the aggregates of Aβ and tau, various β-sheet binding dyes such as congo-red and thioflavin have been isolated as candidate compounds for PET imaging, and their use for visualizing SPs and NFTs in animal models and human subjects has been attempted. Representative and currently available Aβ and tau PET tracers are listed in Table 1. The earliest PET studies identified the capability of [^18^F]FDDNP, a fluorine-modified derivative of the lipophilic compound DDNP, as Aβ PET tracer (Barrio et al., 1999; Small et al., 2006). However, this tracer has not become widely used for *in vivo* imaging because it demonstrates a relatively low signal-to-noise ratio, and it also recognizes NFTs, which makes interpretation difficult (Shoghi-Jadid et al., 2002; Thompson et al., 2009; Smid et al., 2013). A superior Aβ-specific PET tracer termed [^11^C]Pittsburgh Compound-B ([^11^C]PiB) was screened as a derivative of thioflavin T, and it is now the most widely characterized radio ligand with improved sensitivity and high specificity against Aβ aggregate (Klunk et al., 2004). A ^18^F-labeled analog of PiB, termed [^18^F]3′-FPiB (Flutemetamol; Vizamyl, GE Healthcare), was developed for diagnostic application and was approved by the US Food and Drug Administration (Yang et al., 2012). In parallel, other Aβ PET tracers, [^18^F]AV-45 (florbetapir; Amyvid, Eli Lily) and [^18^F]AV-1 (florbetaben; Neuraceq, Piramal Imaging), have also been independently engineered from diaryl alkenes and are currently available for clinical diagnosis (Herholz and Ebmeier, 2011; Vallabhajosula, 2011). For the purpose of standardizing the difference of these Aβ PET tracers, several groups have investigated direct comparison of PET imaging data obtained by sequential scanning using several tracers in the same cohort (Vandenberghe et al., 2010; Wolk et al., 2012; Landau et al., 2013, 2014). As for the cortical retention ratio of [^11^C]PiB, both [^18^F]3′-FPiB and [^18^F]AV-45 demonstrate a highly significant ability to discriminate between control subjects and AD patients, with a high linear correlation to the data obtained by [^11^C]PiB. However, it has been pointed that the retention level in white matter varies among the tracers, which makes it more challenging to precisely measure the cortical retention ratio to the reference brain region such as the brain stem, which contains a large amount of white matter (Landau et al., 2014). Hence, it would still be a worthwhile investment to improve and characterize the capability of Aβ PET tracers (Kudo et al., 2007; Yousefi et al., 2011; Cselenyi et al., 2012). The development race of Tau PET tracers is just heating up (reviewed in Ariza et al., 2015; Villemagne et al., 2015). Three lines of chemical probes have been independently screened and characterized for their potential clinical utility (Table 1). [^18^F]THK523 was originally engineered as a derivative of quinoline, and it demonstrates high affinity and selectivity for tau aggregates in *in vitro* assay (Fodero-Tavoletti et al., 2011). Although human PET imaging using [^18^F]THK523 indicated its specificity not for Aβ but for NFTs, a relatively high background of this radiotracer in white matter was initially anticipated for its further clinical use (Fodero-Tavoletti et al., 2011; Harada et al., 2013). Improved novel radiotracers labeled [^18^F]THK5105, [^18^F]THK5117 and [^18^F]THK5351, 2-arylquinoline derivatives, were recently engineered by the same group and demonstrated superior affinity and specificity in *in vitro* assays, making these tracers promising candidates for tau PET imaging (Okamura et al., 2014; Tago et al., 2014; Villemagne et al., 2014). [^18^F]T807 and [^18^F]T808, also screened as benzimidazole pyrimidine derivatives, demonstrated specific selectivity to tau aggregates with nano-molar affinity (Zhang et al., 2012; Xia et al., 2013). Human PET studies using these radiotracers revealed significant association between clinical severity and their cortical retention, and this observation was also followed by a good correlation with post-mortem pathological NFT lesions (Chien et al., 2013). [^11^C]PBB3 was developed as a phenyl/pyridinyl-butadienyl-benzothiazoles/benzothiazolium (PBB) derivative that possesses superior affinity and specificity for NFTs, much higher than for SPs, by radioautography using brain sections from both rodent models and AD patients (Maruyama et al., 2013). [^11^C]PBB3 PET analysis of AD patients and non-AD tauopathies revealed that the severity of tau accumulation correlates well with clinical scores and follows the pathological staging model proposed by Braak. Because of it being a relatively new topic, as well as the highly competitive nature of this research field, a side-by-side direct comparison of the capability of the respective tau PET tracers in the same individuals has not yet been achieved. However, recent studies based on *in vitro* binding assays indicate that those various tracers demonstrate preferential binding capability to distinctive tau pathologies observed in AD and non-AD, suggesting each tracer potentially recognizes the different structural status of tau aggregates (Maruyama et al., 2013; Marquie et al., 2015). Overall, current tau PET imaging is promising, and detailed characterization and improvement of independent radiotracers will provide better clinical insight and shed greater light on the detection of AD progression.

**Table 1:** **Reference list for Aβ and Tau PET radiotracers**.

**Target**	**Radiotracer for PET imaging of SPs or NFTs**	**Approval for clinical use**	**References**
Aβ/SPs	[^18^F]FDDNP		Barrio et al., 1999; Shoghi-Jadid et al., 2002; Small et al., 2006; Thompson et al., 2009; Smid et al., 2013
Aβ/SPs	[^11^C]PiB		Klunk et al., 2004
Aβ/SPs	[^18^F]3′-FPiB	Flutemetamol; Vizamyl, GE healthcare	Yang et al., 2012
Aβ/SPs	[^18^F]AV-1	Florbetaben; Neuraceq, Piramal imaging	Rowe et al., 2008
Aβ/SPs	[^18^F]AV-45	Florbetapir; Amyvid, Eli Lily	Choi et al., 2009
Tau/NFTs	[^18^F]THK523		Fodero-Tavoletti et al., 2011; Harada et al., 2013
Tau/NFTs	[^18^F]THK5105		Okamura et al., 2014
Tau/NFTs	[^18^F]THK5117		Okamura et al., 2014
Tau/NFTs	[^18^F]THK5351		Villemagne et al., 2014
Tau/NFTs	[^18^F]T807		Xia et al., 2013
Tau/NFTs	[^18^F]T808		Zhang et al., 2012
Tau/NFTs	[^11^C]PBB3		Maruyama et al., 2013

### Non-amyloid PET imaging for detecting disease progression

In addition to Aβ and Tau PET imaging, various disease-associated pathophysiologies such as aberration of energy metabolism, glial inflammation, dysfunction of calcium homeostasis, and imbalanced neuronal activity can also be targeted as potential biomarkers of AD progression. The benefits of characterizing these additional biological parameters for AD diagnosis are the following: (i) accumulating a repertory of biomarkers will eventually contribute to establishing a PET biomarker database that will allow us to conduct more precise diagnoses and case-by-case categorizations with unbiased criteria by the combinations of several parameters; (ii) the signal-to-noise ratio of current amyloid PET imaging is still being developed and is not yet sufficient to detect early stages of pathological lesions; (iii) some of the biomarkers can be useful for the diagnosis of other neurodegenerative disorders as common pathophysiological abnormalities. [^18^F]fluordeoxy glucose ([^18^F]FDG) PET scan has been conducted to identify glucose hypometabolism in AD patients, and it is generally selected as the initial choice for the clinical diagnosis of AD (McGeer et al., 1986; Mosconi, 2005). However, the difficulty in identifying a specific and precise time window of the AD status has been a matter of debate, as [^18^F]FDG hypometabolism reflects various mixture effects including less neuronal excitability and neuronal loss, which are widely observed in various neurodegenerative disorders (Jack and Holtzman, 2013). Brain inflammatory response with reactive gliosis is the other potential target for staging AD progression (Heppner et al., 2015). A considerable amount of evidence has indicated that activated reactive astrocytes contribute to clearing SPs in the early stage of AD. Since the inflammation is massively amplified by the paracrine neuroprotective response, it would be one of the key candidates as an early-stage biomarker that can detect subtle changes indicating pathological abnormality. In fact, using a tau transgenic mouse model, it was demonstrated that microglial activation proceeds to NFTs formation (Yoshiyama et al., 2007), suggesting its potential utility as a much earlier-stage diagnostic biomarker. This evidence supports the concept of the potential utility of several inflammation-related molecules for early clinical diagnosis. On the other hand, it has been debated whether inflammatory response represents both aspects of neuroprotective and neurodegenerative signals (Higuchi et al., 2010; Heneka et al., 2015; Heppner et al., 2015). Particularly, there is evidence that prolonged microglial activation accelerates the neurodegenerative process (Maeda et al., 2007; Ji et al., 2008), suggesting a potential difficulty for interpreting inflammatory parameters as early-stage biomarkers. In any case, it should be feasible to target a molecule that can segregate astrocytic and microglial activations. Based on this concept, TSPO (Translocator Protein 18 kDa, also known as Peripheral Benzodiazepine Receptor; PBR) has been targeted for imaging nueroinflammation (reviewed in Jacobs et al., 2012). Since increased TSPO expression in activated microglia has been associated with a number of neurological diseases (reviewed in Venneti et al., 2006), several PET tracers for TSPO (e.g.,; [^11^C]*(R)*-PK11195, [^11^C]PBR28, [^11^C]DAA1106, [^11^C]AC5216, and [^18^F]DPA-714) have been studied to visualize the microglial activation in animal models and AD patients (Jacobs et al., 2012; Mori et al., 2012; Golla et al., 2015). However, there are several limitations of current TSPO ligands such as selectivity to microglia and different affinity in polymorphisms (reviewed in Venneti et al., 2013; Turkheimer et al., 2015). Regardless, imaging neuroinflammation will offer one of diagnostic tools for neurological diseases.

### Pitfalls of PET imaging

PET imaging is an extremely powerful tool for the purpose of non-invasive clinical diagnosis. However, it is still not easy to install these imaging systems into a single laboratory; it would be a costly and space-consuming challenge to establish a special radiation control facility requiring a high level of expertise for the maintenance and manipulation of radioactive molecules. In addition, there are also technical limitations in regard to spatial and temporal resolution, and there is not yet a suitable approach for evaluating binding specificity and molecular dynamics of the tracer, pivotal criteria for reliable imaging interpretation. For instance, despite the development of newly screened radiotracer candidates based on considerable effort and time for synthesis and radiolabeling, *in vitro* and *ex vivo* binding assays such as autoradiography under low magnification are recognized as so far being the sole method for verifying the capability of the tracer. An additional weakness of PET technique is the inability to obtain new biological insight into the structural and/or environmental status of target molecules, meaning that the solid radiation signal we monitor during PET scans completely lacks that kind of molecular-based information. Therefore, an ideal approach would be to evaluate the biological validity and capability of the tracer by complementary technologies side-by-side, and this would comprehensively enhance the developmental productivity by mutual complementary and synergistic effect of different technologies. In the next section, we will discuss the utility of fluorescence imaging in the effort to lessen the gap in PET imaging.

## Fluorescence imaging to dissect molecular pathophysiology

### Utility of fluorescence for *in vivo* brain imaging

During the rapid evolution of optical microscope technologies over the last several decades, fluorescence imaging has become a powerful tool for current modern neuroscience (Giepmans et al., 2006; Wilt et al., 2009). A variety of microscope systems with combinations of many available fluorescence probes supply a wide range of applications including single molecular analysis, live cell imaging, and conventional histological analysis in animal brain. In particular, the recent availability of the multi-photon excitation technique has broken down the limitation of optical scattering in thick tissues and expanded the utility of fluorescence for *in vivo* brain imaging in animal models (reviewed in Svoboda and Yasuda, 2006). In this system, only target fluorophores in a restricted volume can be excited by simultaneous photon absorption using a long-wavelength pulse laser, allowing us to illuminate molecular dynamics up to a depth of ~1 mm. To conduct a long period of efficient excitation and detection (at least 1 month), setting of a chronic cranial window onto the bone of the skull is generally used (Tomita et al., 2005). Furthermore, current progress of fluorescence-based microendoscopy using optical fibers has enabled us to conduct cellular level imaging within the deep areas of brain tissue such as hippocampus and striatum in freely moving animals (reviewed in Mehta et al., 2004; Oh et al., 2013). In spite of mechanical inflexibility and physical invasiveness caused by surgical insertion of submillimeter-diameter fiber optic devices, it offers potential applications for longitudinal studies of disease progression (Barretto et al., 2011). At this stage, these approaches are specifically limited to usage in rodent models and is technically still far removed from any availability for human diagnosis. However, the combinatorial use of the AD mouse model with multi-color fluorescence probes should represent a beneficial approach for the monitoring of the progressive course of the AD cascade simultaneously, relying on fast temporal kinetics and high spatial resolution.

### *In vivo* fluorescence imaging for detecting Aβ and tau pathologies

As most of the β-sheet binding chemical compounds engineered for PET radiotracers possess their own fluorescence derived from a polycyclic aromatic hydrocarbon backbone structure, these small chemical compounds have ideal multimodalities for side-by-side *in vivo* imaging comparison by both fluorescence and PET techniques (Hawe et al., 2008). So far, studies focusing on the beneficial usage of *in vivo* fluorescence approaches have been published from only a few laboratories (Table 2). As pioneer studies, SPs in APP transgenic mouse, which demonstrates massive Aβ pathology, were first successfully visualized by the hydrophilic chemical compound Thioflavin S or by anti-Aβ antibody conjugated to fluorescein by two-photon microscopy (Bacskai et al., 2001; Christie et al., 2001). Since these reagents cannot penetrate BBB, the authors injected them directly into the imaging area of the brain surface, demonstrating that the size and morphology of SPs are rapidly cleared by immunization with Aβ peptide during the course of a few days (Bacskai et al., 2001). The relatively hydrophobic Congo-Red derivative Methoxy-X04 was next developed and used to visualize Aβ pathology in brain of APP transgenic mouse by intraperitoneal bolus administration (Klunk et al., 2002). In addition, importantly, it was also verified that Pittsburgh Compound-B (PiB) successfully visualized SPs in mouse brain parenchyma by intravenous administration, opening a new possibility for the simultaneous characterization of a chemical tracer for both fluorescence and PET imaging (Bacskai et al., 2003). The advantage of these compounds is their ability to pass through BBB and therefore provide very important information on the kinetics and tissue distribution of Aβ pathology with great spatiotemporal resolution (~μm for spatial and ~sec for temporal). Although initial studies have suggested a relatively stable appearance of existing SPs over several months, more recent studies indicated that both SPs and cerebral amyloid angiopathy (CAA), the cerebrovascular Aβ deposition in arterial vessels, grow at a consistent rate (Robbins et al., 2006; Garcia-Alloza et al., 2007; Hefendehl et al., 2011). It has also been demonstrated that newly developed SPs grow rapidly for 24 h and dynamically change their morphology, suggesting that steady-state Aβ pathology undergoes flexible regulation by various environmental factors in living animal. Similar approaches to visualize tau pathology in Tau transgenic mouse model have also been used. By direct application of Thioflavin S, NFTs with neuronal loss in brain of rTg4510 tau transgenic mice were first evaluated by multi-photon microscopy (Spires-Jones et al., 2008). X-34, a Congo-Red derivative, and PBB3 were next independently developed by different groups and used for successful visualization of NFTs with great spatiotemporal resolution by intravenous injection in living mouse models (de Calignon et al., 2010; Maruyama et al., 2013). Although the detailed *in vivo* time course of NFTs formation has not yet been well-characterized, these studies promise the dual utility of the compounds for both radiological and fluorescence *in vivo* imaging applications. Importantly, both local abnormalities of Aβ and Tau pathology can be detected by multi-photon excitation at a very early stage, indicating that the fluorescence approach has a greater advantage for identifying beneficial molecular clues linking early pathological change in AD model animals.

**Table 2:** **Reference list for *in vivo* fluorescence imaging in living mouse model**.

**Mouse model**	**Reagents and application for *in vivo* visualization of SPs or NFTs**	**Fluorescence reporters for *in vivo* visualization of AD pathophysiology**	**References**
PDAPP (APP V717F)	Thioflavin S, anti-Ab w/Fluorescein (intracerebral injection)		Bacskai et al., 2001
Tg2576 (APPsw)	Thioflavin S, anti-Ab (10D5) conjugated Cy3 (intracerebral injection)		Christie et al., 2001
PS-APP (PS1M146L vs. Tg2576)	Methoxy-X04 (i.p.)		Klunk et al., 2002
PDAPP Tg2576 PS-APP	Thioflavin T, Thioflavin S, PiB (i.v.)		Bacskai et al., 2003
Tg2576	Methoxy-X04 (i.p.)		Robbins et al., 2006
PS1dEx9/APPsw	curcumin (i.v.) Methoxy-X04 (i.p.)		Garcia-Alloza et al., 2007
PS1L166P/APPsw	Methoxy-X04 (i.p.)		Hefendehl et al., 2011
3xTg-AD APPdutch/iowa Tg2576	–	fluo-4 AM	Takano et al., 2007
PS1dEx9/APPsw	Methoxy-X04 (i.p.)	YC3.60 camereon (AAV, intracerebral injection)	Kuchibhotla et al., 2008
PS45(PS1G384A)/APP23(sw)	Thioflavin S (intracerebral injection)	Oregon green BAPTA	Busche et al., 2008
PS1dEx9/APPsw	Methoxy-X04 (i.p.)	Oregon green BAPTA SR-101 for astrocyte	Kuchibhotla et al., 2009
PS45/APP23	Thioflavin S (intracerebral injection)	Fluo-8 AM	Busche et al., 2012
PS1dEx9/APPsw	Methoxy-X04 (i.p.)	CaMKII 5′UTR-EGFP/Venus-3'UTR (AAV, intracerebral injection)	Meyer-Luehmann et al., 2009
PS1dEx9/APPsw	Methoxy-X04 (i.p.)	Crossbreeding to Arc/dVenus mice	Rudinskiy et al., 2012
Tg2576	Methoxy-X04 (i.p.)	Crossbreeding to B6C3-YFP mice Crossbreeding to Cx3CR1/GFP mice	Meyer-Luehmann et al., 2008
PDAPP	Methoxy-X04 (i.p.)	Crossbreeding to Cx3CR1 mice	Koenigsknecht-Talboo et al., 2008
rTg4510 (Tau P301L)	Thioflavin S (intracerebral injection)	FLICA (intracerebral injection)	Spires-Jones et al., 2008
rTg4510 (Tau P301L)	Thiopflavin S (intracerebral injection) X-34 (i.v.)	FLICA (intracerebral injection)	de Calignon et al., 2010
PS19 (Tau P301S)	PBB3 (i.p., i.v.)		Maruyama et al., 2013
rTg4510 (Tau P301L)	Thioflavin S (intracerebral injection)	YC3.60 camereon (AAV, intracerebral injection)	Kopeikina et al., 2013
rTg4510 (Tau P301L)	Thioflavin S (intracerebral injection)	YC3.60 camereon (AAV, intracerebral injection)	Kuchibhotla et al., 2014

### Utility of fluorescence biosensors of AD pathophysiology

Recently, unique functional fluorescence biosensors to monitor biophysical properties were developed for dissecting the detailed physiological status of animal brain. Since Green fluorescent protein (GFP) was discovered from jellyfish *Aequorea victoria* by Shimomura et al., many unique native fluorecent proteins emitting diverse colors have been isolated from multiple marine organisms (Shimomura et al., 1962). The improved fluorescent proteins in terms of wavelength, brightness, photostability, and less oligomeric property were successfully engineered by random mutagenesis analysis, and the fluorescent protein color palette has been extensively matured from blue to a near-infrared spectrum (Giepmans et al., 2006). Engineered fluorescent proteins with enhanced unique original properties (e.g., photo activation/conversion, environment sensitivity, etc. …) have also been created during this process, opening a new window to the utility of fluorescent proteins as molecular biosensors generally termed “genetically encoded fluorescence indicators” (GEFIs) to monitor a wide variety of biological parameters including calcium, voltage, ATP, cAMP, glutamate, and kinase activities (Knopfel, 2012; Miyawaki and Niino, 2015). In addition, approaches to the use of virus-mediated gene delivery or conventional animal genetics with specific promoter-dependent regulation allow us to manipulate specific subpopulations of neurons or glial cells, and GEFIs are certain to become a powerful and broadly used tool for dissecting the molecular pathophysiology of neurodegenerative disorders.

With the combination of fluorescence reporters and biosensors, attempts have been made to illuminate the more complicated AD-related pathophysiology by multi-photon excitation techniques (Table 2). Abnormal dendritic curvature and spine loss associated with SPs in brain of APP transgenic mouse were first illuminated in living neurons expressing GFP by either Adeno associated virus (AAV)-mediated manipulation or by crossbreeding with fluorescence reporter mouse (Spires et al., 2005). Using AAV-mediated overexpression of YC3.6 calcium sensor proteins, Aβ pathology-related aberrant overloading of neuronal calcium in the somatosensory cortex was then detected with great temporal resolution (Kuchibhotla et al., 2008). Interestingly, local administration of Oregon-Green BAPTA (OGB), a small-molecule calcium dye, also revealed hyperactivity in neurons and disrupted synchronous calcium oscillation in the astrocyte network associated with Aβ pathology (Busche et al., 2008; Kuchibhotla et al., 2009). In addition, inhibition of Ca^2+^/calmodulin-dependent protein phosphatase 2B (Calcineurin) attenuated the calcium dysregulation and structural abnormality of neurites. These findings suggest that dysfunction of calcium homeostasis is one of the key environmental parameters related to amyloid pathology. In addition to these observations, cross-breeding of APP transgenic mice with Cx3CR1 mice, which specifically express GFP in microglial cells, identified robust microglial activation associated with Aβ pathology (Meyer-Luehmann et al., 2008). Importantly, this immune response has been constantly observed during both the development of SPs and anti-Aβ immunization, suggesting that inflammation plays an essential role in the regulation of the dynamics of Aβ pathology (Koenigsknecht-Talboo et al., 2008). As an additional unique fluorescence reporter, transgenic mouse expressing destabilized Venus under control of immediate early gene Arc promoter was generated to identify disrupted orchestrated neuronal network activity in an APP transgenic mouse model (Rudinskiy et al., 2012). Taken together, these studies strongly indicate the power of the fluorescence system in combination with the current genetics approaches to provide major insights into the AD pathophysiology. Using the fluorescence indicator of caspase activation (FLICA), it was also demonstrated that the majority of caspase-positive neurons contain NFTs in brain of rTg4510 tau transgenic mouse (Spires-Jones et al., 2008). Interestingly, infrequent NFTs-negative neurons demonstrated rapid suppression of caspase activity followed by NFTs formation within 1 day (de Calignon et al., 2010). This indicates that NFTs formation potentially has a neuroprotective role against caspase-mediated neuronal apoptosis. The other surprising observation was that rTg4510 tau transgenic mouse demonstrated normal calcium dynamics despite massive synaptic loss in both sensory and visual cortex, suggesting that tau-related synaptic abnormality was induced by a calcium-independent mechanism (Kopeikina et al., 2013; Kuchibhotla et al., 2014). Dissection of the brain status of tau transgenic mouse model by *in vivo* fluorescence imaging is still at a developmental stage, and therefore additional studies with fluorescence reporter can be expected to further expand the future potential and contribution of the examination of the tau-related pathophysiology of AD.

### Limitations of fluorescence imaging

As we summarize in this section, fluorescence-based optic imaging has become an attractive approach to simultaneously monitor the interaction between target molecules and disease-related pathophysiology with high spatiotemporal kinetics, and to offer solid molecular-based insight for dissecting the *in vivo* mechanism underlying the progression of the neurodegenerative process in AD model animals. It also offers a relatively simple and inexpensive system that can be relatively easy to install into laboratory space, and potentially provide powerful biomedical applications such as *in vivo* high-throughput drug screening. However, considering future *in vivo* applications, there are still many technical challenges and space that will require solutions. First, the issue of optical diffusion tissue *in vivo* still strongly restricts the visualization of fluorophore in only the surface area even in mouse brain (reviewed in Svoboda and Yasuda, 2006). Second, the potential invasiveness and toxicity caused by both the surgery process and local genetic manipulation limit the usage of these techniques in animals, although not in human (reviewed in Hillman, 2007). Third, it is also important to note that overexpression of fluorescent reporter proteins potentially has undesirable photostability, toxicity, and mislocalization (reviewed in Shaner et al., 2005). Based on an understanding of these advantages and disadvantages, we will then consider how to integrate each of the imaging modalities in the future as described in the last section.

## Perspectives for future imaging multimodality

### PET imaging for early detection of AD pathology

The current successful establishment of Aβ and tau PET radiotracers in combination with the available biomarkers promises the availability of PET imaging for the clinical diagnosis of AD. The next challenge for future PET neuroimaging is the design of more advanced chemical probes that specifically recognize the distinct conformational and/or modification status of aggregates. In fact, it is known that PiB efficiently detects SPs in AD brain but not in mouse brain, suggesting that a modification process in different biological species can influence the *in vivo* binding capability of the tracer (Klunk et al., 2005). Recent studies suggest that not filamentous but soluble oligomeric aggregates of Aβ and tau are toxic species during the pathological course of AD (Cheng et al., 2013). In particular, tau protein undergoes multiple post-translational modifications such as phosphorylation, nitration, or truncation, which are predicted to associate with the pathogenic mechanism(s) of AD (Marcus and Schachter, 2011). Using *in vitro* aggregation assay, several groups have successfully identified unique chemical compounds that preferentially bind to intermediate aggregated species of Aβ and tau (Wischik et al., 1996; Bolognesi et al., 2010; Smith et al., 2010; Carrasco-Gallardo et al., 2012; Jameson and Dzyuba, 2013; Teoh et al., 2015). More recently, pentameric formyl thiophene acetic acid (pFTAA) was screened to successfully visualize the conformation specific status of tau aggregates in living dorsal root ganglion neurons isolated from tau transgenic mouse model (Brelstaff et al., 2015). Therefore, some chemical compounds that potentially have specific binding affinity to toxic oligomer or modified species of Aβ and tau might be more suitable tracers for the early detection of AD pathology. Various intermediates of tau aggregates have been isolated during the pathological course of NFTs formation, and it has been highly debated whether soluble oligomeric aggregates of tau are toxic species (Makrides et al., 2003; Maeda et al., 2006; Sahara et al., 2007; Peterson et al., 2008; Lasagna-Reeves et al., 2010; Bader et al., 2011; Patterson et al., 2011). It has also been hypothesized that the formation process of intracellular tau inclusion potentially has a trophic role against tau toxicity (Spires-Jones et al., 2011; Cowan and Mudher, 2013). In this regard, to segregate the early pathological tau species that are toxic vs. those that are more neuroprotective would be a more challenging, but also a more attractive investment for advanced preclinical diagnosis. A fluorescence-based imaging approach will have a great advantage in addressing this fundamental issue. Current elegant studies have also demonstrated a cell-based fluorescence system that can detect the very early stage of tau aggregates isolated from juvenile tau transgenic mouse brain by fluorescence resonance energy transfer (FRET), pointing out its potential complementary application as a more physiological drug screening system (Holmes et al., 2014). Taken together, it is clearly important to expand multimodal imaging approaches to characterize the basic capability of chemical compounds, and this will eventually improve the screening efficiency and open attractive directions for more sophisticated molecular diagnoses with appropriate data interpretation.

### Genetic manipulation of animal models

To follow this multimodal approach, a key component would be advancing technical innovation for genetic manipulation. Many transgenic/knock-in mouse models overexpressing mutant APP, PSs, and/or Tau have been established during the last two decades, and a tremendous amount of fundamental knowledge about the AD cascade has been acquired. However, these classical genetic systems still have many limitations, as follows. First, it is quite difficult to reproduce the spatiotemporal dynamics of the AD pathological progression in these classical models because transgene overexpression is controlled by specific promoter activity. Second, it is still expensive and time-consuming to generate mouse lines, and technical innovation to improve productivity will be required. Third, rodent models may in fact represent the ultimate limitation to the reproduction of the human brain circumstances because of the species barrier. As an advanced mouse model, a drug inducible Cre-loxP system, or a Tet on/off system, has been incorporated into mouse genetics to achieve more precise conditional control of transgene expression (Santacruz et al., 2005). In recent studies, P301L mutant Tau was strictly expressed in Layer II/III neurons in the entorhinal cortex by neuropsin promoter in combination with a tetracycline inducible system (de Calignon et al., 2012; Liu et al., 2012). An interesting finding was that this region-specific expression of tau causes progressive spreading of NFTs pathology along the tri-synaptic entorhinal-hippocampus circuit in an anterograde manner, suggesting the presence of toxic tau species that can be transmitted via trans-synaptic communication. This prion-like propagation theory is further supported by studies with *in vivo* administration of fibrillar tau aggregates into mouse brains resulting in the spread of tau pathology to synaptically connected distant brain regions (Clavaguera et al., 2009, 2013; Lasagna-Reeves et al., 2012; Iba et al., 2013). As an alternative approach, the AAV system has become a widely used technique to achieve spatiotemporal manipulation of transgene expression. In fact, circuit-dependent pathological spreading of tau pathology was also recently achieved by local administration of AAV-encoding mutant tau into the entorhinal cortex of adult rodent (Siman et al., 2013). Therefore, a very fascinating approach would be to generate a tau mouse model genetically modified in the local cell population of a specific neuronal circuit, and to trace the *in vivo* spatiotemporal consequence of tau propagation in the brain of the same individual living animal by fluorescence and PET simultaneously, which would potentially facilitate further understanding of non-cell autonomous toxic mechanisms shared among various neurodegenerative disorders.

Very recently, an artificial nuclease-mediated gene edition presented as the CRISPR/CAS9 system has rapidly expanded its utility for the generation of animal models from insects to a primate system (Sander and Joung, 2014). Using such innovative tools, the establishment of genetic imaging reporter animals is one of the attractive directions for future AD research. Several studies have demonstrated that herpes simplex virus type 1 thymidine kinase (HSV1-TK)-mediated phosphorylation and intracellular accumulation of nucleoside analogs such as 9-(4-fluoro-3-hydroxymethylbutyl) guanine (FHBG) is a useful PET reporter system for assessing gene expression, protein-protein interactions, and enzymatic activity (Gambhir et al., 2000; Luker et al., 2002; Massoud et al., 2010). Current advances in chemical biology are also creating the potential availability of multiple new technologies such as advanced affinity tag and light manipulation for various *in vivo* imaging applications (Kramer et al., 2013). Therefore, the combination of these genetic manipulations is certain to contribute to the efficient development of advanced animal model systems, and will allow us to conduct simultaneous multimodal imaging comparisons to monitor the sequential pathophysiology of AD progression.

## Concluding remarks

Multimodal neuroimaging may open an attractive direction for future translational research, and allow us to simultaneously monitor the actual behavior of target molecules of interest in living subjects from a microscopic to a macroscopic view. Based on the current progress of PET and the fluorescence system, there are still a lot of areas and issues to be addressed, and integration of these very different techniques will still present some challenging hurdles. At this moment, all we need is to sincerely evaluate both the advantages and disadvantages of individual imaging systems, and to consider ways to complement these very different techniques by seeking even small improvements in a step-by-step manner. Eventually, this steady work and effort will result in a breakthrough in the area of advanced biomedical research including future diagnostics.

## Author contributions

Researching the data for the article: MS and NS. Drafting of the manuscript: MS. Discussion of the content: MS, MH, TS, and NS. Review and editing of the manuscript: MH, TS, and NS.

### Conflict of interest statement

The authors declare that the research was conducted in the absence of any commercial or financial relationships that could be construed as a potential conflict of interest.
